# Vector-Host Interactions of *Culiseta melanura* in a Focus of Eastern Equine Encephalitis Virus Activity in Southeastern Virginia

**DOI:** 10.1371/journal.pone.0136743

**Published:** 2015-09-01

**Authors:** Goudarz Molaei, Philip M. Armstrong, Charles F. Abadam, Karen I. Akaratovic, Jay P. Kiser, Theodore G. Andreadis

**Affiliations:** 1 Center for Vector Biology & Zoonotic Diseases, The Connecticut Agricultural Experiment Station, New Haven, Connecticut, United States of America; 2 Suffolk Mosquito Control, Department of Public Works, Suffolk, Virginia, United States of America; University of Texas Medical Branch, UNITED STATES

## Abstract

Eastern equine encephalitis virus (EEEV) causes a highly pathogenic mosquito-borne zoonosis that is responsible for sporadic outbreaks of severe illness in humans and equines in the eastern USA. *Culiseta (Cs*.*) melanura* is the primary vector of EEEV in most geographic regions but its feeding patterns on specific avian and mammalian hosts are largely unknown in the mid-Atlantic region. The objectives of our study were to: 1) identify avian hosts of *Cs*. *melanura* and evaluate their potential role in enzootic amplification of EEEV, 2) assess spatial and temporal patterns of virus activity during a season of intense virus transmission, and 3) investigate the potential role of *Cs*. *melanura* in epidemic/epizootic transmission of EEEV to humans and equines. Accordingly, we collected mosquitoes at 55 sites in Suffolk, Virginia in 2013, and identified the source of blood meals in engorged mosquitoes by nucleotide sequencing PCR products of the mitochondrial *cytochrome b* gene. We also examined field-collected mosquitoes for evidence of infection with EEEV using Vector Test, cell culture, and PCR. Analysis of 188 engorged *Cs*. *melanura* sampled from April through October 2013 indicated that 95.2%, 4.3%, and 0.5% obtained blood meals from avian, mammalian, and reptilian hosts, respectively. American Robin was the most frequently identified host for *Cs*. *melanura* (42.6% of blood meals) followed by Northern Cardinal (16.0%), European Starling (11.2%), Carolina Wren (4.3%), and Common Grackle (4.3%). EEEV was detected in 106 mosquito pools of *Cs*. *melanura*, and the number of virus positive pools peaked in late July with 22 positive pools and a Maximum Likelihood Estimation (MLE) infection rate of 4.46 per 1,000 mosquitoes. Our findings highlight the importance of *Cs*. *melanura* as a regional EEEV vector based on frequent feeding on virus-competent bird species. A small proportion of blood meals acquired from mammalian hosts suggests the possibility that this species may occasionally contribute to epidemic/epizootic transmission of EEEV.

## Introduction

Eastern equine encephalitis virus (EEEV) (*Togaviridae*, *Alphavirus*) is a highly pathogenic mosquito-borne virus that occurs in discrete foci in the eastern USA. Human and equine cases are concentrated in the Atlantic and Gulf Coast states in proximity to freshwater hardwood swamps where the primary mosquito vector [*Culiseta (Cs*.*) melanura*] breeds [1–2]. EEEV is amplified in a bird-mosquito transmission cycle that depends on frequent feeding of *Cs*. *melanura* on virus-competent bird species [3–7]. Humans and horses become infected when mosquitoes act as bridge vectors by feeding opportunistically on viremic birds and then mammalian hosts. *Cs*. *melanura* is considered an unlikely bridge vector because it feeds mainly on birds [2, 8]. Recent analyses of *Cs*. *melanura* sampled from the northeastern USA revealed that 1–11% of blood meals were obtained from mammals including equines and humans [5–7, 9], suggesting that this species may occasionally transmit EEEV to horses and possibly humans.

The mid-Atlantic region remains an important focal area for EEEV transmission since the initial discovery of the virus in 1933 during an equine epizootic in Virginia and neighboring states [10]. A number of freshwater swamp complexes are located throughout this region that support dense populations of *Cs*. *melanura* mosquitoes and large aggregations of migratory and permanent bird species [11–13]. This includes the Great Dismal Swamp and surrounding communities, which constitute an established EEEV focus in southeastern Virginia [14]. Residential and commercial developments have fragmented much of the swamp perimeter, leaving large pockets of swamp habitat interspersed with human populations [15]. This landscape provides ideal conditions for human infection by bringing virus-infected mosquitoes from swamp habitats into close proximity with residential areas. An earlier investigation in southeastern Virginia found that *Cs*. *melanura* comprised >95% of EEEV-positive mosquito pools during a five-year surveillance period [14]. Despite its importance as a vector, the blood-feeding patterns of *Cs*. *melanura* have not been evaluated in this region by molecular methods. Such analyses are critical to identify specific avian hosts involved in enzootic cycling of virus and to evaluate the potential for *Cs*. *melanura* to act as an occasional vector of EEEV to mammalian hosts.

In this study, we investigated the transmission cycle of EEEV in southeastern Virginia by determining the host-feeding patterns of *Cs*. *melanura* during a season of intense virus transmission. Specifically, blood-fed *Cs*. *melanura* were collected at sites between May-October 2013 in Suffolk, Virginia adjacent to the Great Dismal Swamp. Vertebrate blood meals were identified by polymerase chain reaction (PCR) amplification and sequencing of the mitochondrial *cytochrome b* gene. In addition, host-seeking female mosquitoes were collected and tested for EEEV infection to evaluate spatial and temporal patterns of virus transmission. Our findings highlight the importance of *Cs*. *melanura* as a regional EEEV vector based on frequent feeding on virus-competent bird species with occasional blood meals from mammalian hosts.

## Materials and Methods

### Ethics Statement

Eight of the mosquito trapping sites were located in the Great Dismal Swamp National Wildlife Refuge (GDSNWR). A specific permission (permit # R2013-07) to collect and test mosquitoes from these sites was obtained from the GDSNWR. The remaining 47 trapping sites were located on properties owned or operated by the City of Suffolk, VA. Because some of the of the coauthors are employed by the City of Suffolk Public Works Department, they were authorized to collect and test mosquitoes on these properties, and no specific permissions were required for these locations/activities. The field studies associated with this project did not involve endangered or protected species.

### Study area

**Fig 1:**
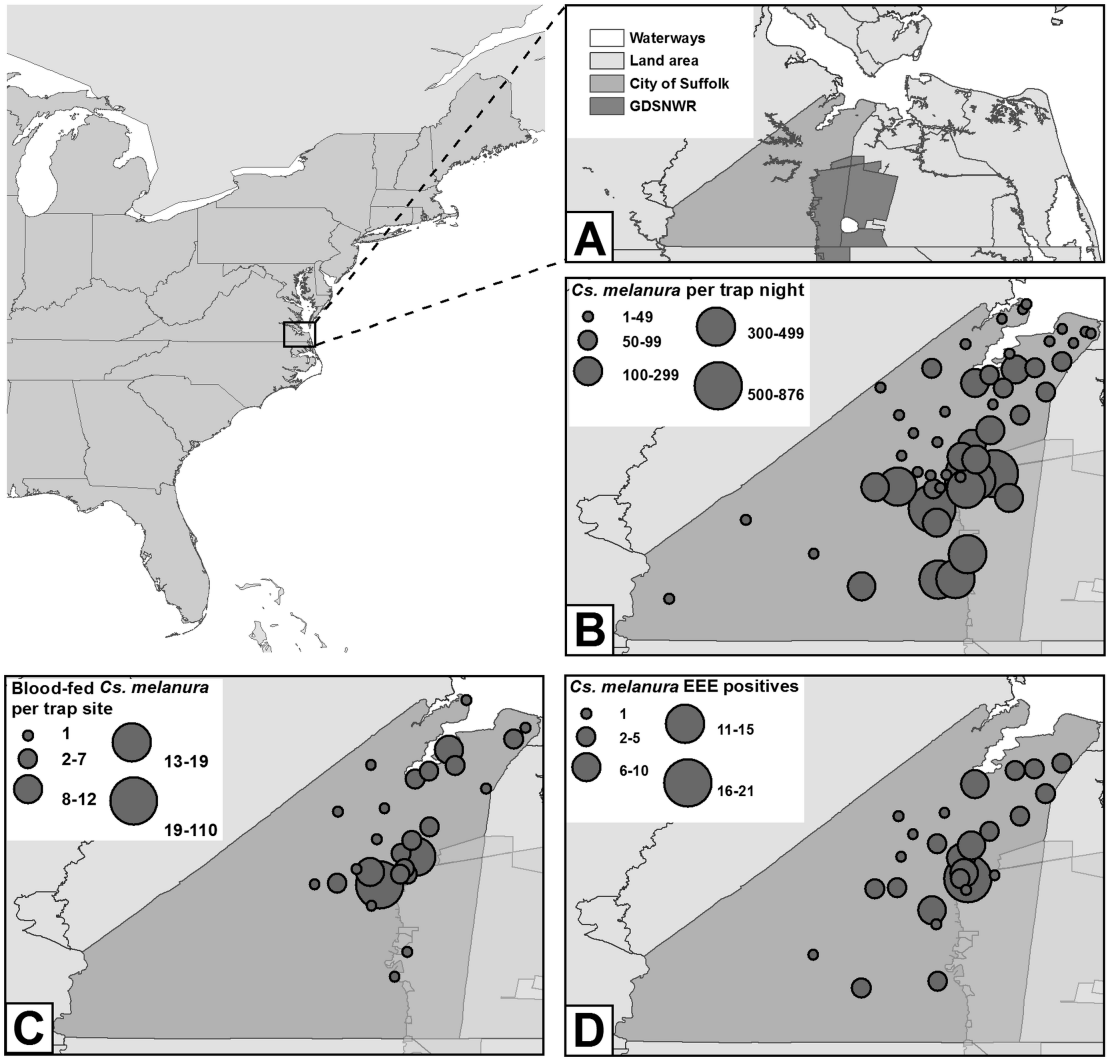
Map of the study area and mosquito collection sites in Suffolk, VA, 2013. (A) Close-up of Hampton Roads in southeastern Virginia with the City of Suffolk and Great Dismal Swamp National Wildlife Refuge shaded in. (B) Average number of *Cs*. *melanura* captured at each trap site per trap night. (C) Total number of blood-fed mosquitoes collected at each trap site. (D) Total number of eastern equine encephalitis virus-positive *Cs*. *melanura* pools at each trap site.

City of Suffolk is part of a larger region called Hampton Roads located in southeastern Virginia. Suffolk is located at 36°44′ 29″ N 76° 36′ 36″ W approximately 50 km west of the Atlantic Ocean and 15 km south of the Chesapeake Bay. The city has a land area of 1,036 km^2^ with a human population of >85,000. This makes Suffolk the largest city in Virginia by land area with the penultimate human population density of Virginia’s independent cities. The city is comprised of the Chowan River and the James River watersheds, situated within the upland and lowland coastal plain provinces with an elevation range of sea level to 110 feet. Approximately 59% of the land area is zoned as agriculture, 26% as mixed urban, suburban, and commercial, and 15% as conservation. The agricultural and conservation land is covered in or interspersed with freshwater hardwood swamps. The GDSNWR is the largest portion of the conservation area with 148 km^2^ within Suffolk’s borders (Fig 1). One-third of the refuge is located in the southeastern portion of the city; the remaining two-thirds extend into the City of Chesapeake, Virginia and the counties of Gates, Camden, and Pasquotank in North Carolina. In the northeast and central parts of the city, urban areas are adjacent to swamp environments. This unique variety of habitats provides an opportune and diverse milieu for potential interactions between mosquito vectors of arboviruses and their prospective vertebrate hosts.

### Mosquito Collection

Mosquitoes were collected from April through October 2013 at 55 sites in Suffolk using four different trap methods. BG-Sentinel traps (Biogents, Regensburg, Germany) were baited with Biogents human skin non-toxic chemical lures and carbon dioxide from a gas cylinder. CDC light traps (BioQuip Products, Rancho Dominguez, CA), and CDC light traps attached to modified CDC-Collection Bottle Rotators (BioQuip Products) were baited with carbon dioxide from a gas cylinder. A modified design of a Reiter gravid trap [16] was self-constructed and baited with a mixture of chicken manure, alfalfa, yeast, and water that fermented for seven to fourteen days. Most traps were placed in areas that consisted of residential or commercial land, bordered by woodland or swamp habitats. Mosquito trapping at each site was performed weekly with the exception of GDSNWR sites where traps were set intermittently as time allowed. Traps were set between 12:00 PM and 3:00 PM and picked up the next morning between 6:30 AM and 9:30 AM.

### Mosquito specimen processing and morphological identification

Trap chambers with live mosquitoes were collected from field sites and transported to the City of Suffolk Mosquito Control (CSMC) laboratory and sedated with triethylamine, with the exception of specimens collected in the CDC-Collection Bottle Rotator traps in which a dichlorvos compound was used. Specimens were then transferred to glass petri dishes for species identification by morphological characters using a dissecting microscope and regional taxonomic key [17]. *Cs*. *melanura* specimens with visible blood meals were separated, placed in labeled microcentrifuge tubes, and stored in a -18°C freezer until they were shipped on dry ice to the Connecticut Agricultural Experiment Station (CAES) for host-blood meal analyses and virus testing. Starting in June, the non-blood-fed *Cs*. *melanura* were pooled and vialed into groups of approximately 50 or less individuals. For most trap sites a maximum of 10 *Cs*. *melanura* pools/week were tested at CSMC for EEEV and West Nile virus (WNV) using Vector Test (Vector Test Systems, Thousand Oaks, CA). The remaining pools were vialed and stored at -18°C for potential PCR testing. Roughly 40 of these additional pools were selected each week and shipped on dry ice to the CAES for virus testing.

### DNA isolation from blood-fed mosquitoes

DNA was isolated from the abdomen of the blood-fed mosquitoes by using DNAzol BD (Molecular Research Center, Cincinnati, OH) according to the manufacturer’s recommendation, with modifications as described previously [5–7, 18]. Briefly, blood-fed mosquitoes were placed on new microscope slides under a dissecting microscope and abdomens were removed with clean razor blades to avoid cross-contamination. Individual mosquito abdomens were homogenized by a micropestle (USA Scientific, Ocala, FL) in a 1.5 mL microcentrifuge tube containing 400 DNA-zol BD. Following addition of 15 μL proteinase K (Qiagen, Valencia, CA) the homogenate was mixed and incubated at 70°C for 10 min, and then centrifuged at 18,000 x g for 10 min. The supernatant was transferred to a new microcentrifuge tube and DNA was precipitated by adding 200 μL of isopropanol or ethanol and 3–4 μL Poly Acryl Carrier (Molecular Research Center) and incubating at room temperature for 10 min. The tube containing precipitated DNA was centrifuged at 3,200 x g for 6 min, and then supernatant was discarded. The DNA pellet was then washed twice with 75% ethanol, air-dried briefly, reconstituted in TE buffer (10 mmol/L Tris-HCl, pH 8.0, 1 mmol/L EDTA) and stored at –20°C for further analysis.

### Mosquito blood meal identification

Isolated DNA from the mosquito blood meals was used as DNA templates in PCR reactions to screen for the vertebrate host choice. Primers for PCR screening were based on the mitochondrial *cytochrome b* gene to identify avian and mammalian host species. PCR reactions were initially performed with avian- and mammalian-specific primer pairs, according to previously described protocols [5–7, 18]. Sequences for avian-specific primer pairs were 5’-GAC TGT GAC AAA ATC CCN TTC CA-3’ (forward) and 5’-GGT CTT CAT CTY HGG YTT ACA AGA C-3’ (reverse), with an anticipated amplified product size of 508 bp. Sequences for mammalian-specific primers were 5’-CGA AGC TTG ATA TGA AAA ACC ATC GTT G-3’ (forward) and 5’-TGT AGT TRT CWG GGT CHC CTA-3’ (reverse), with an anticipated amplified product size of 772 bp. The TaqPCR core kit (Qiagen) was used for PCR reactions in accordance with the manufacturer’s recommendation. PCR reaction mix and thermal-cycling conditions were as described in earlier studies [6, 18]. The primer set used to screen for mammalian hosts in the present study has also amplified reptilian and amphibian *cytochrome b* genes in our earlier investigation [19]. The GeneAmp PCR System 9700 or Veriti Dx Thermal Cycler (Applied Biosystems, Foster City, CA) were utilized for PCR cycling. PCR- amplified products were purified using QIAquick PCR purification kit (Qiagen) and sequencing of both DNA strands was carried out on 3730xL DNA Analyzers, along with Big Dye chemistries (Applied Biosystems) at the Keck Sequencing Facility, Yale University, New Haven, CT. Sequences were analyzed and annotated using ChromasPro version 1.7.5 (Technelysium Pty Ltd., Tewantin, Australia) and identified by comparison to the GenBank DNA sequence database (National Center for Biotechnology Information, available online: www.ncbi.nlm.nih.gov/blast/Blast.cgi).

### Virus testing of mosquitoes

Cell culture and PCR testing of mosquitoes were performed at the CAES. The head and thorax of blood-fed mosquitoes were tested individually whereas non-blood-fed mosquitoes were combined into pools of 50 or less. Mosquitoes were placed in 2 mL microcentrifuge tubes containing a copper BB and 1 mL PBS-G (phosphate buffered saline, 30% heat-inactivated rabbit serum, 0.5% gelatin, and 1 x antibiotic/antimycotic) was added to each tube. Samples were homogenized for 4 min at 25 cycles per sec on a Vibration Mill MM300 (Retsch Laboratory, Irvine, CA) and then centrifuged at 4°C for 10 min at 4,500 x g. The supernatant (100 μL) was inoculated onto a monolayer of Vero cells growing in a 25 cm^2^ flask. Cells were maintained at 37°C in 5% CO_2_ and examined daily for cytopathic effect (CPE) on days 3–7 post-inoculation. RNA was extracted from CPE-positive cell cultures and the corresponding mosquito pool using the viral RNA Kit (Qiagen), and tested by real-time RT-PCR for EEEV and WNV [20–21]. Samples were also screened for Highlands J virus (HJV) by real-time RT-PCR using in-house primers and probe: HJ-E1 5’-ACT TCC GAG GTA ACG TGG TG-3’ (forward), HJ-E1 5’-GCG ACA CTG CAC AGC ATT AT-3’ (reverse), and HJ-E1probe (FAM-TGT CTA GCG GCA CGT GGC ATT ATG-BHQ) and the same thermal cycling conditions described for WNV [21].

Vector Test in-house assays were conducted at CSMC and tested *Cs*. *melanura* pools for WNV and EEEV. Protocols used for the in-house testing followed a modified version of the alternative procedure outlined in the Vector Test WNV and EEEV antigen panel assay instruction manual (Medical Analysis Systems, Camarillo, CA). These tests were typically performed the day of or the day after specimens were collected in the field. Specimens were stored at 4°C if any delay of five hours or more occurred between collecting and testing. Mosquitoes designated for in-house testing were placed in a 2 mL microcentrifuge tube containing one 4.5 mm zinc-plated steel BB and 1750μL of Vector Test grinding solution. Samples were then homogenized using the TissueLyser II (Qiagen) at a frequency of 30 oscillations per sec for 6 min and subsequently centrifuged at 6,000 x g for 90 sec. A 250 μL aliquot of the supernatant was pipetted out of each sample and transferred to a corresponding new 2 mL microcentrifuge tube. A single Vector Test dipstick was placed in each tube containing the supernatant. Following 15 min in the supernatant, the dipsticks were removed, placed on a paper towel to dry, and the results were read and recorded. The remaining portions of the samples correlating to the positive strips were stored at -18°C until they were shipped on dry ice to Pennsylvania Department of Health Bureau of Laboratories (PDHBL) for PCR confirmation. A small, random selection of negative Vector Test samples was sent for PCR confirmation as a control. Samples were shipped to PDHBL within two weeks of in-house testing. Mosquito pools that were identified as positive for WNV or EEEV via the Vector Test in-house assay were only considered positive after PCR confirmation.

### Avian population abundance estimates

**Fig 2:**
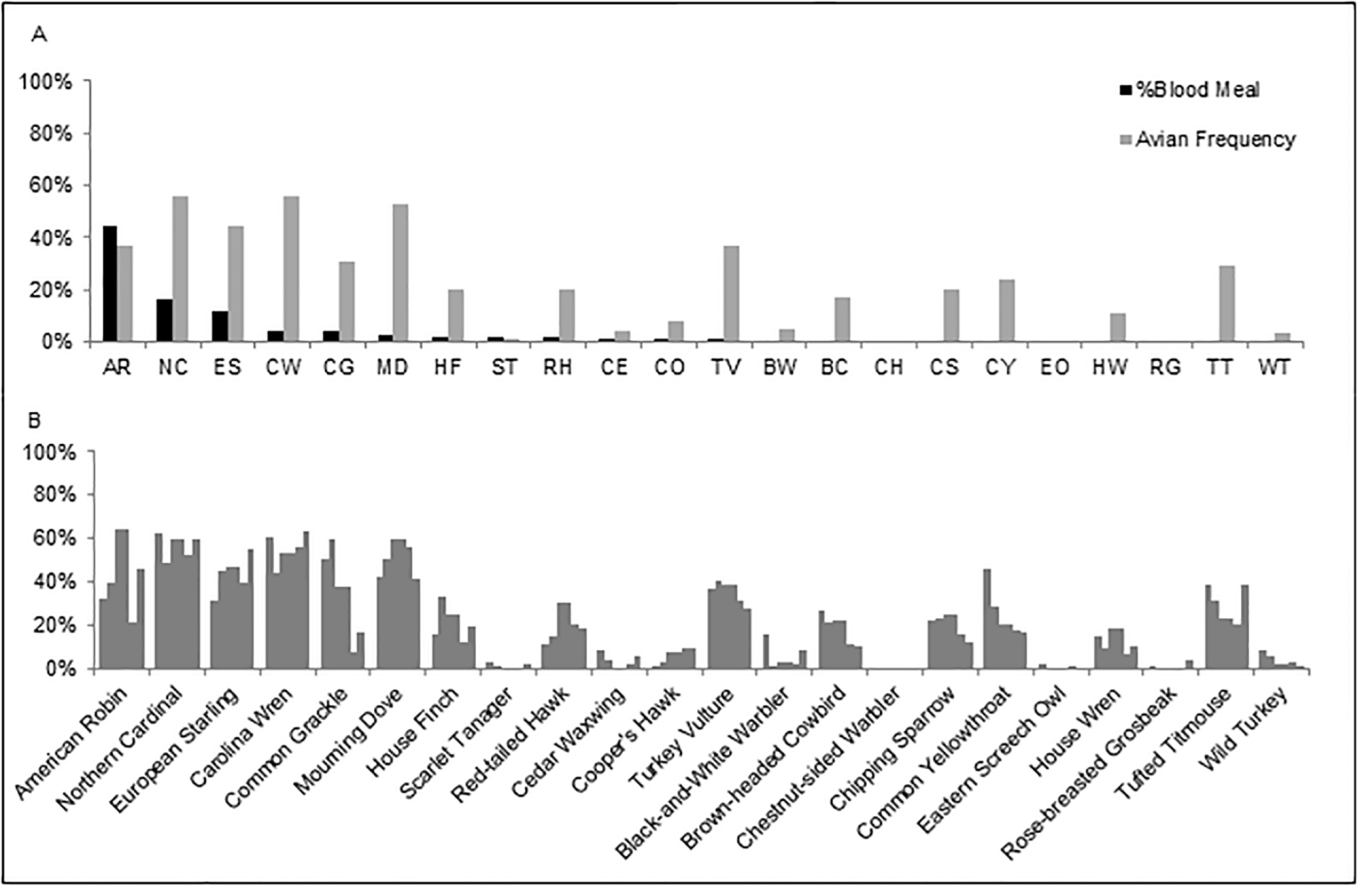
Avian-derived blood meals in *Culiseta melanura* and avian frequencies. (A) Percentage of avian-derived blood meals in *Cs*. *melanura* compared with the average avian frequencies in the City of Suffolk, VA and surrounding cities/counties (City of Portsmouth, City of Chesapeake, Southampton County, and Isle of Wight County), May through October 2013. (B) Monthly frequencies of avian species based on point count data in the City of Suffolk, VA and surrounding cities/counties (City of Portsmouth, City of Chesapeake, Southampton County, and Isle of Wight County), May through October 2013.

Frequency estimates of regional avian species (Fig 2) were obtained from eBird, a program developed by the Cornell Laboratory of Ornithology and the National Audubon Society to track bird abundance and distribution in North America. Estimates were obtained via checklist data from recreational and professional birdwatchers and were accessed through the World Wide Web (http://www.ebird.org). The term “frequency” refers to the proportion of checklists that reported a species within a particular date range and region. The frequency data in this publication consist of information collected on a monthly basis from May through October 2013 for the City of Suffolk and surrounding cities and counties.

## Results

### Mosquito species composition and abundance

**Fig 3:**
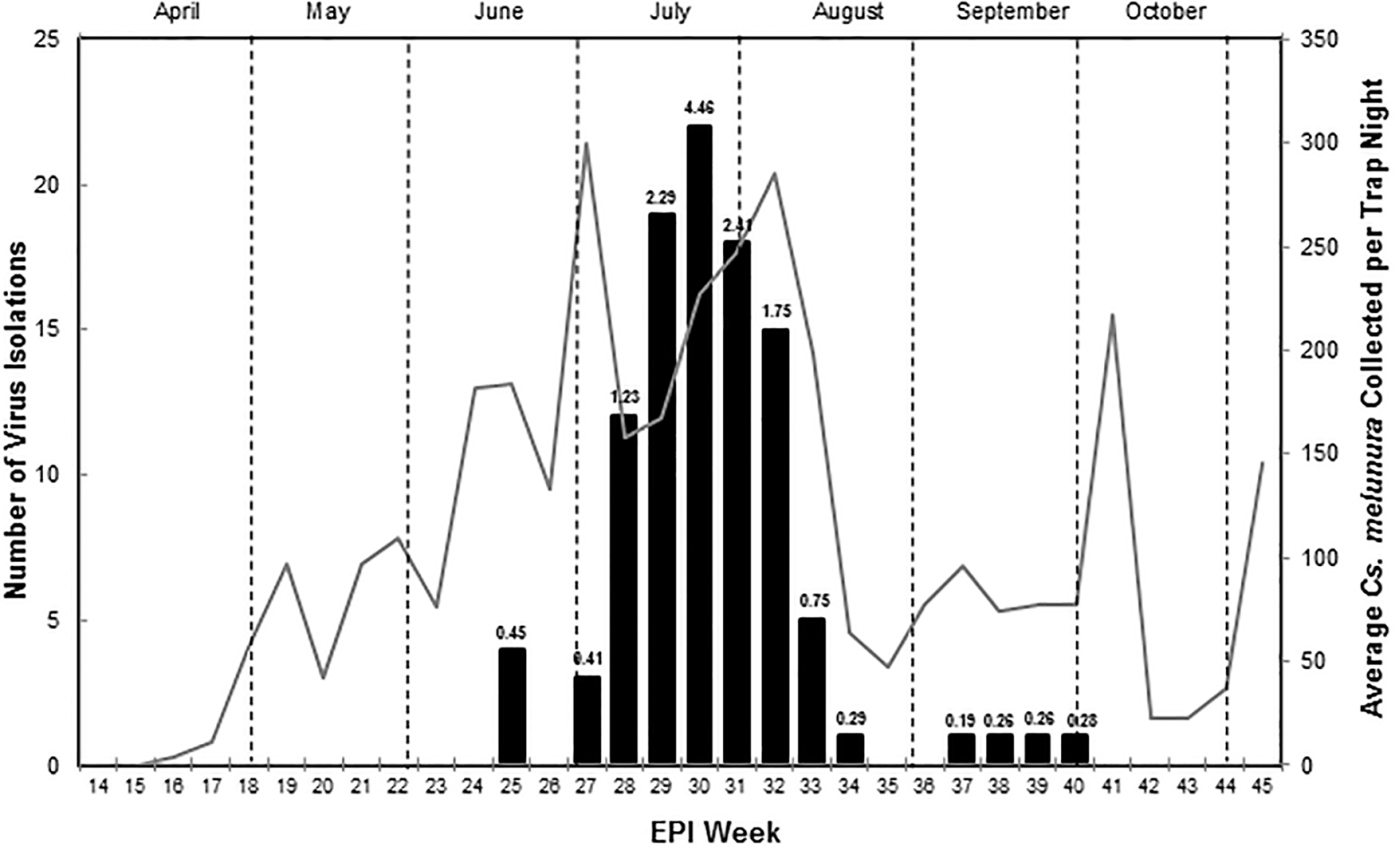
Eastern equine encephalitis virus (EEEV) isolations and Maximum Likelihood Estimations (MLEs) in *Culiseta melanura*. Weekly EEEV isolations and MLEs compared to average *Cs*. *melanura* collected per trap night in Suffolk VA, 2013. The line graph represents the weekly average *Cs*. *melanura* captured per trap citywide. The bar graph represents the total number of EEEV-positive mosquito pools from each week with a corresponding MLE above the bar (calculated with both Vector Test and PCR positives of *Cs*. *melanura* pools).

During 2013, a total of 283,218 mosquitoes were collected over 1,597 trap nights from 55 sites throughout the City of Suffolk. Collections were comprised of 62.0% *Cs*. *melanura* (Figs 1B and 3), 7.0% *Culex* (*Cx*.) *salinarius*, 6.7%, *Coquillettidia perturbans*, 5.9% *Cx*. *erraticus*, 3.3% *Aedes albopictus*, 3.1% *Ochlerotatus canadensis*, and less than 3% of each of the remaining 26 species (Table 1). A total of 211 blood-fed *Cs*. *melanura* were collected between April 25 and October 17, 2013, from 27 out of 55 trap sites throughout the City of Suffolk (Fig 1C).

**Table 1:** Number and percentage of mosquitoes collected from April through October 2013 in Suffolk, VA.

Mosquito species	No.	% of Total
*Culiseta melanura*	175518	62.0
*Culex salinarius*	19910	7.0
*Coquillettidia perturbans*	18919	6.7
*Culex erraticus*	16621	5.9
*Aedes albopictus*	9375	3.3
*Ochlerotatus canadensis*	8872	3.1
*Anopheles crucians*	6051	2.1
*Psorophora ferox*	6003	2.1
*Ochlerotatus atlanticus*	4098	1.4
*Culex pipiens*	4051	1.4
*Culex restuans*	2873	1.0
*Psorophora columbiae*	2835	1.0
*Uranotaenia sapphirina*	2393	0.8
*Aedes vexans*	2088	0.7
*Anopheles quadrimaculatus*	1601	0.6
*Ochlerotatus triseriatus*	658	0.2
*Anopheles punctipennis*	285	0.1
*Ochlerotatus infirmatus*	263	0.1
*Psorophora howardii*	119	<0.1
*Ochlerotatus japonicus*	98	<0.1
*Culex territans*	72	<0.1
*Orthopodomyia signifera*	58	<0.1
*Psorophora ciliata*	39	<0.1
*Ochlerotatus sollicitans*	23	<0.1
*Ochlerotatus taeniorhynchus*	23	<0.1
*Ochlerotatus cantator*	10	<0.1
*Toxorhynchites rutilus septentrionalis*	4	<0.1
*Ochlerotatus sticticus*	2	<0.1
*Ochlerotatus fulvus pallens*	1	<0.1
*Ochlerotatus mitchellae*	1	<0.1
*Ochlerotatus thibaulti*	1	<0.1
*Psorophora mathesoni*	1	<0.1
Damaged-Unidentifiable specimens*	352	0.1
**Total**	283218	100

* Damaged-Unidentifiable, indicates specimens that were not able to be identified to species by morphological characteristics due to severe damage from environmental conditions and/or trapping equipment.

### Vertebrate host choices of *Cs*. *melanura*

Vertebrate hosts of 188 engorged *Cs*. *melanura* collected from an active EEEV focus in Suffolk, VA were successfully identified to species level. Of which, 179 (95.2%), 8 (4.3%), and 1 (0.5%) had avian, mammalian, and reptilian origins, respectively (Table 2). We identified 22 avian species in 15 families and 5 orders as hosts for *Cs*. *melanura*. American Robin was the most frequent source of blood meal comprising 42.6% (N = 80) of all vertebrate-derived blood meals, followed by Northern Cardinal 16.0% (N = 30), European Starling 11.2% (N = 21), Carolina Wren and Common Grackle, each 4.3% (N = 8), and then 17 other avian species comprising 16.8% (N = 32) of all blood meals. Four mammalian species representing four families and two orders served as hosts for *Cs*. *melanura*, including Domestic Dog 1.6% (N = 3), Domestic Cat and Raccoon, each 1.1% (N = 2), and White-tailed Deer 0.5% (N = 1). We also identified one (0.5%) Common Box Turtle as a reptilian host for *Cs*. *melanura*.

**Table 2:** Number and percentage of blood meals identified from *Cs*. *melanura* collected from an active focus of eastern equine encephalitis virus activity in Suffolk, Virginia, 2013.

Host Common Name	Host Scientific Name	Family (Order)	Residency Code	No.	% of Total
**Avian**					
American Robin	*Turdus migratorius*	Turdidae (Passeriformes)	P	80	42.6
Northern Cardinal	*Cardinalis cardinalis*	Cardinalidae (Passeriformes)	P	30	16
European Starling	*Sturnus vulgaris*	Sturnidae (Passeriformes)	P	21	11.2
Carolina Wren	*Thryothorus ludovicianus*	Troglodytidae (Passeriformes)	P	8	4.3
Common Grackle	*Quiscalus quiscula*	Icteridae (Passeriformes)	P	8	4.3
Mourning Dove	*Zenaida macroura*	Columbidae (Columbiformes)	P	5	2.7
House Finch	*Haemorhous mexicanus*	Fringillidae (Passeriformes)	P	4	2.1
Scarlet Tanager	*Piranga olivacea*	Cardinalidae (Passeriformes)	T	4	2.1
Red-tailed Hawk	*Buteo jamaicensis*	Accipitridae (Accipitriformes)	P	3	1.6
Cedar Waxwing	*Bombycilla cedrorum*	Bombycillidae (Passeriformes)	W, P	2	1.1
Cooper's Hawk	*Accipiter cooperii*	Accipitridae (Accipitriformes)	W, P	2	1.1
Turkey Vulture	*Cathartes aura*	Cathartidae (Accipitriformes)	P	2	1.1
Black-and-white Warbler	*Mniotilta varia*	Parulidae (Passeriformes)	S	1	0.5
Brown-headed Cowbird	*Molothrus ater*	Icteridae (Passeriformes)	P	1	0.5
Chestnut-sided Warbler	*Setophaga pensylvanica*	Parulidae (Passeriformes)	T	1	0.5
Chipping Sparrow	*Spizella passerina*	Emberizidae (Passeriformes)	P	1	0.5
Common Yellowthroat	*Geothlypis trichas*	Parulidae (Passeriformes)	P, S	1	0.5
Eastern Screech-Owl	*Megascops asio*	Strigidae (Strigiformes)	P	1	0.5
House Wren	*Troglodytes aedon*	Troglodytidae (Passeriformes)	S, P	1	0.5
Rose-breasted Grosbeak	*Pheucticus ludovicianus*	Cardinalidae (Passeriformes)	T	1	0.5
Tufted Titmouse	*Baeolophus bicolor*	Paridae (Passeriformes)	P	1	0.5
Wild Turkey	*Meleagris gallopavo*	Phasianidae (Galliformes)	P	1	0.5
**Mammalian**					
Domestic Dog	*Canis lupus familiaris*	Canidae (Carnivora)		3	1.6
Domestic Cat	*Felis catus*	Felidae (Carnivora)		2	1.1
Raccoon	*Procyon lotor*	Procyonidae (Carnivora)		2	1.1
White-tailed Deer	*Odocoileus virginianus*	Cervidae (Artiodactyla)		1	0.5
**Reptilian**					
Common Box Turtle	*Terrapene carolina*	Emydidae (Testudines)		1	0.5
**Total**				**188**	

Residency Codes: P, permanent resident (found year round in the region); S, summer resident (present in the region during the nesting season); T, transient (migrates through the region in the spring and/or fall); W, winter (present in the region during the winter season).

### Seasonal variation in avian host composition

Monthly prevalence of *Cs*. *melanura* blood meals acquired from five avian species individually, and the remaining birds collectively, is shown in Table 3. In May, we identified 90 avian-derived blood meals, of which 54.4% (N = 49) were from American Robin, followed by European Starling 15.6% (N = 14), Northern Cardinal 10.0% (N = 9), Common Grackle 6.7% (N = 6), and then other bird species 13.3% (N = 12). In June, frequency of blood meals from American Robins declined to 26.7% (N = 4), whereas other bird species constituted 73.3% of all blood meals (N = 14). Notably, in June, Carolina Wren served as the most frequent source of blood meals (33.3%, N = 5). In July, the frequency of blood meals from American Robins reached 50.0% (N = 14). Northern Cardinals constituted 66.7% (N = 14), and 33.3%, (N = 2) of blood meals in August and September, respectively. In October, 57.9% (N = 11) of the avian-derived blood meals were from American Robins. Chi squared test showed that there is a highly significant temporal difference in the proportion of blood meals from American Robin and Northern Cardinal (p<0.0001). This indicates that *Cs*. *melanura* obtained a majority of blood meals earlier in the season, and from Northern Cardinals and other bird species later in the season. We also performed Smirnov test [22] for temporal difference between American Robin and Northern Cardinal as hosts for *Cs*. *melanura*. The fractional difference between the two time courses indicates that frequency of blood meals from Northern Cardinal peaks after that of the American Robin (p< 0.005).

**Table 3:** Monthly frequencies of avian-derived blood meals of *Cs*. *melanura* collected from an active focus of eastern equine encephalitis virus activity in Suffolk, Virginia, 2013.

Avian Host	May n (%)	June n (%)	July n (%)	August n (%)	September n (%)	October n (%)	Total
American Robin	49 (54.4)	4 (26.7)	14 (50.0)	1 (4.8)	1 (16.7)	11 (57.9)	80
Northern Cardinal	9 (10.0)	1 (6.7)	2 (7.1)	14 (66.7)	2 (33.3)	2 (10.5)	30
European Starling	14 (15.6)	2 (13.3)	2 (7.1)	1 (4.8)	1 (16.7)	1 (5.3)	21
Carolina Wren	-	5 (33.3)	2 (7.1)	1 (4.8)	-	-	8
Common Grackle	6 (6.7)	1 (6.7)	-	1 (4.8)	-	-	8
Other species	12 (13.3)	2(13.3)	8(28.6)	3(14.3)	2(33.3)	5(26.3)	32
**Total**	**90**	**15**	**28**	**21**	**6**	**19**	**179**

### Avian population analysis

Monthly frequency analyses of avian species in the City of Suffolk and surrounding cities and counties is depicted in Fig 2. Relatively higher abundances of American Robin, Northern Cardinal, European Starling, Carolina Wren, and Mourning Dove were noticed throughout the year. The percentage of *Cs*. *melanura* that acquired blood meals from these bird species were as expected based on their abundance. However, for other avian species, such as Turkey Vulture, Tufted Titmouse, and a few other birds, it was considerably lower than expected based on their frequency estimates.

### Eastern equine encephalitis virus infection in mosquitoes

To estimate the prevalence of EEEV infection in *Cs melanura*, a total of 120,476 field-collected mosquitoes were separated into 2,608 mosquito pools and tested for viral infection. EEEV was detected in 106 mosquito pools of *Cs*. *melanura* from mid-June to early October (Fig 3). Of which, 64 were detected using Vector Test with a 100% RT-PCR positive confirmation rate and 4 were detected from 24 randomly selected Vector Test negatives, resulting in a 17% RT-PCR false-positive confirmation rate. The number of virus-positive mosquito pools peaked during the fourth week of July with 22 positive pools and a MLE infection rate of 4.46 per 1,000 mosquitoes. EEEV-positive mosquito pools declined sharply during August and were detected infrequently during September and October. EEEV was detected in 26 trapping locations with focal areas located in and around GDSNWR (Fig 1). In addition, five *Cs*. *melanura* pools tested positive for WNV and two mosquito pools contained HJV. None of the blood-fed mosquitoes tested positive for arboviral infection.

## Discussion

This study provides further insight into the ecology of EEEV by analyzing the host-feeding patterns of *Cs*. *melanura* in an active focus of virus transmission in the mid-Atlantic region of the USA. Our results confirm that *Cs*. *melanura* feeds primarily on passerine birds with a focus on American Robin, Northern Cardinal, European Starling, Carolina Wren, and Common Grackle in this region of the USA. Identification of 95.2% of *Cs*. *melanura* blood meals from avian hosts is in agreement with the results of recent studies in the northeastern USA. The percentage of avian-derived blood meals comprised 89.6% in Connecticut [5], 89.7% in New Jersey [9], 94.2% in New York [6], and 99.0% in Massachusetts [7]. Studies conducted in the southeastern USA- Tennessee, Alabama and Florida- have also reported close interactions of *Cs*. *melanura* with Northern Cardinal, Carolina Wren, American Robin, and several other avian species as preferred hosts [23–25]. A small proportion of *Cs*. *melanura* (4.8%) obtained blood meals from mammalian and reptilian species, suggesting occasional contribution of this species to epidemic/epizootic transmission of EEEV to incidental hosts including humans and equines, in addition to its prominent role in enzootic virus transmission among birds.

Recent studies have used molecular techniques to analyze mosquito blood meals to consistently identify American Robin as a frequent host for *Cs*. *melanura* [5–7, 9, 18]. As a common tree-roosting bird, permanent and temporary migrating populations of American Robin are active in woodland habitats including open and forested urban/suburban and rural settings, riparian forests, and early successional forests [26–29]. Identification of American Robin as the most frequent (N = 80, 42.6%) host for *Cs*. *melanura* in the present study is consistent with the results of earlier blood meal analyses in the northeastern USA, where 21.7% (N = 115) of all blood meals in Massachusetts [7]; 22.9% (N = 11) in Connecticut [5]; and 9.1% (N = 46) in New York [6] were from this bird species. In a laboratory analysis, American Robin has been identified as a competent amplification host for EEEV [30]. EEEV has also been isolated from American Robins collected in Massachusetts [31] and New Jersey [32].

Furthermore, serosurveys indicate that this bird species is frequently exposed to EEEV in several studies from the eastern USA [31–35]. Thus, close interactions of *Cs*. *melanura* with American Robins in the present study suggest the importance of these birds in early amplification and maintenance of EEEV throughout the season in Virginia.

Northern Cardinals served as the second most frequent source of blood meals for *Cs*. *melanura* in the present study. Northern Cardinals have a widespread distribution in mid-Atlantic, southeast, and Gulf regions, and are found in a wide variety of habitats such as woodland edges, thickets, and gardens in urban/suburban settings. Reservoir competence of Northern Cardinals as hosts for EEEV, determined by the duration of viremia and the proportion of *Cs*. *melanura* that become infected following feeding on this bird species, was moderate [30]. Serologic evidence of EEEV infection in Northern Cardinals has been reported from an active virus focus in central New York [36], and from Cape May County in New Jersey [32]. Furthermore, EEEV has been isolated from Northern Cardinals captured from the Atlantic seaboard of the USA [37].

European Starlings were identified among frequent hosts for *Cs*. *melanura* in our study. Since their introduction in the nineteenth century, European Starlings have become one of the most numerous songbirds with year-round activity in North America. European Starlings are closely associated with human dwellings and are commonly found in city streets and agricultural fields. Because European Starlings are not native to North America, infection with EEEV could be more severe with sudden onset, a condition that may change the reservoir competence of these birds [30]. In a laboratory study to compare the intensity and duration of viremia in European Starlings and American Robins, following infection by *Cs*. *melanura* bite, the intensity of viremia was greatest on the first day following infection. The intensity of viremia in European Starlings (10^7.3^ pfu/mL) was greater than that of American Robins (10^5.7^ pfu/mL). The intensity of viremia in European Starlings remained sufficiently high to infect mosquitoes for three days, in contrast to only one day for American Robins [30].

Carolina Wren was also identified as one of the frequent hosts for *Cs*. *melanura* in our study. Carolina Wrens are permanent residents in their range of distribution in mid-Atlantic, southeast, and Gulf coast regions. However, their range of distribution may extend north of the breeding range, especially in the fall. They are active in brush piles and low tangles in woods and backyards in the southeast. In an investigation of EEEV in relation to the avian community of a coastal cedar swamp in Cape May County, New Jersey, antibody prevalence was nearly 40% in Carolina Wren [32].

Common Grackle also served as a frequent source of blood meals for *Cs*. *melanura*. Natural habitats for Common Grackles include open woodland, forest edge, grassland, meadows, swamps, marshes, and palmetto hammocks. Reservoir competence of Common Grackle was comparable to that of Northern Cardinals after challenging with EEEV [30]. The presence of antibody against EEEV has been documented in Common Grackles captured from Cape May County, New Jersey [32], and upstate New York [38].

In this study, we found that host-feeding patterns of *Cs*. *melanura* changed during the course of the virus transmission season. There was pronounced feeding on American Robins during May through July and again later in October; however, the intensity of feeding later shifted towards other species such as Northern Cardinals (Table 3). This shift coincided with the build-up of EEEV infection in *Cs*. *melanura* that occurred mostly during July through early-August (Fig 3). Earlier studies also indicate a trend toward temporal diversity of avian hosts for *Cs*. *melanura* throughout the season [4], and a seasonal shift from American Robins towards other birds [7]. Collectively, these findings further implicate American Robins as important amplification hosts for EEEV in a number of geographic regions.

Occasional feeding of *Cs*. *melanura* on mammalian hosts including humans and equines has also been reported from Connecticut [5], New Jersey [9], New York [6], Massachusetts [4, 7], and recently from Vermont [Molaei et al. unpublished data]; where up to 11% of blood meals were from mammals. An earlier study conducted at the Pocomoke Swamp of Maryland neighboring Virginia reported that 12.7% (N = 1,556) of *Cs*. *melanura* blood meals were obtained from mammalian species [39].

## Conclusion

We find that *Cs*. *melanura* mosquitoes feed on a diversity of bird species in southeastern Virginia but focus their feeding on just a few virus-competent bird species during the peak of EEEV amplification from July to early August. *Cs*. *melanura* is a competent vector of EEEV and high virus titers and infection rates in this species have been reported [40–42]. These findings in conjunction with identification of a small proportion of blood meals from mammalian hosts suggest the possibility that *Cs*. *melanura* may occasionally contribute to epidemic/epizootic transmission of EEEV in the region, although this has not been demonstrated.
